# Sleeplessness

**Published:** 1875-12

**Authors:** 


SLEEPLESSNESS.—Dr. George Johnson, in a lecture on the effects of overwork and mental anxiety, alludes to the sleeplessness and anorexia which are almost invariably caused by these conditions. He says it is not always easy to determine whether the loss of appetite is a direct result of the nervous excitement, or whether the restlessness is a result of the diminished supply of nutriment to the brain; but that it is probable that the two conditions have a mutual influence upon each other. In cases resulting from overwork, he has observed in numberless instances that a man who has been more or less restless for many months, and who, during that time, has had a loathing for food, after taking a grain of opium at bedtime for a few nights, sleeps soundly for several hours, and then wakes with an appetite. This tends to prove that in such cases the derangements of the nervous system are first in order of time and importance.

In the cases of delirium tremens the reverse is true; the delirium, wakefulness, and other evidences of nervous disorder are directly due to malnutrition of the brain, and, in spite of all treatment by drugs, will continue until a certain amount of nutriment has been absorbed. In these cases the substitution of narcotics for food has often been attended with rapidly fatal results. We see, then, that in delirium *a potu*, sleep follows, and is favored by taking food; in cases previously nervous, the soporific effect of opium or chloral procures sleep, and then restores the appetite and assists digestion.—*The Lancet.*

